# ^1^H-MR spectroscopy for analysis of cardiac lipid and creatine metabolism

**DOI:** 10.1007/s10741-012-9341-z

**Published:** 2012-09-04

**Authors:** Kiterie M. E. Faller, Craig A. Lygate, Stefan Neubauer, Jürgen E. Schneider

**Affiliations:** Department of Cardiovascular Medicine, British Heart Foundation Experimental Magnetic Resonance Unit (BMRU), Wellcome Trust Centre for Human Genetics, University of Oxford, Roosevelt Drive, Oxford, OX3 7BN UK

**Keywords:** Heart, Metabolism, Magnetic resonance spectroscopy, ¹H-MRS, Creatine, Lipids

## Abstract

Magnetic resonance spectroscopy (MRS) is the only non-invasive, non-radiation-based technique for investigating the metabolism of living tissue. MRS of protons (^1^H-MRS), which provides the highest sensitivity of all MR-visible nuclei, is a method capable of detecting and quantifying specific cardiac biomolecules, such as lipids and creatine in normal and diseased hearts in both animal models and humans. This can be used to study mechanisms of heart failure development in a longitudinal manner, for example, the potential contribution of myocardial lipid accumulation in the context of ageing and obesity. Similarly, quantifying creatine levels provides insight into the energy storage and buffering capacity in the heart. Creatine depletion is consistently observed in heart failure independent of aetiology, but its contribution to pathophysiology remains a matter of debate. These and other questions can in theory be answered with cardiac MRS, but fundamental technical challenges have limited its use. The metabolites studied with MRS are much lower concentration than water protons, requiring methods to suppress the dominant water signal and resulting in larger voxel sizes and longer scan times compared to MRI. However, recent technical advances in MR hardware and software have facilitated the application of ^1^H-MRS in humans and animal models of heart disease as detailed in this review.

## Introduction

Heart failure, defined as the inability of the heart to supply sufficient blood flow to meet the needs of the body, is a major societal burden due to its high prevalence, poor prognosis and cost. In the United Kingdom, it affects at least one person out of 100, and this number is anticipated to rise in the next few years [1]. Despite intensive work, the pathogenesis of this multifactorial syndrome—which includes changes in metabolism—is still poorly understood.

Every day, the heart consumes—in the form of adenosine triphosphate (ATP)—more energy relative to its weight than any other organ [2–4]. To meet its energy demand, a healthy heart predominantly oxidises free fatty acids (FFA) and glucose.

A long-standing hypothesis suggests that the failing heart is energy-starved. However, until the past two decades, this concept—and cardiac metabolism in general—has not really been accessible, mostly due to the lack of techniques to assess metabolic processes of the whole heart in vivo. The advent of surgically and genetically modified (small) animal models, combined with the development of more sophisticated research tools, has sparked a renewed interest in cardiac (energy) metabolism (see for example [5]).

Cardiac Magnetic Resonance (CMR) Imaging has become a clinically well-established tool to assess cardiac morphology, function and viability. However, metabolic processes are not accessible to this technique. Conversely, Magnetic Resonance Spectroscopy (MRS) is a technique that allows non-invasive biochemical analysis of living tissue. Unlike other imaging modalities such as PET or SPECT, it has the combined advantages of inherently providing molecular information, being free of ionising radiation, and not requiring administration of external tracers or contrast agents.

**Fig. 1:**
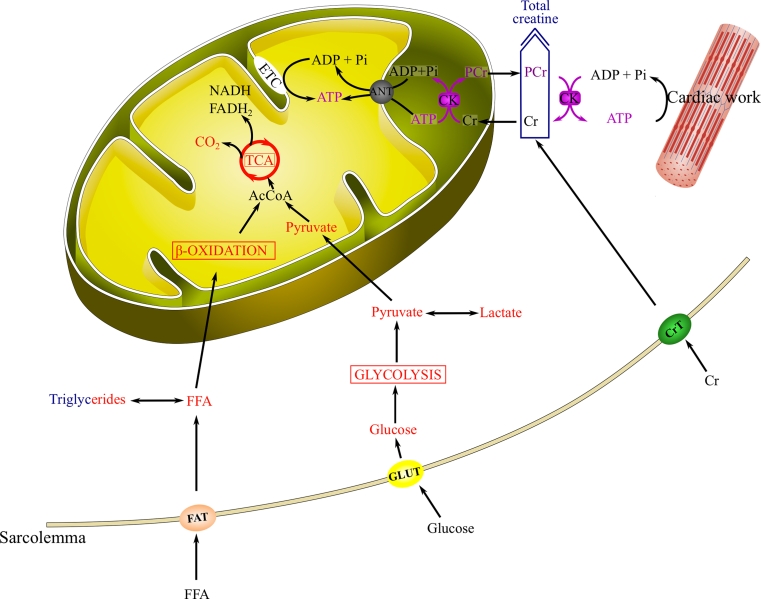
Schematic representation of a myocyte representing the various aspects of cardiac metabolism that are accessible to MRS. Metabolites and flux measured by ^13^C-MRS are highlighted in* red*; by ^31^P-MRS, in* purple*; and by ^1^H-MRS, in* blue*, respectively. *FFA* free fatty acids; *FAT* fatty acid transporter; *GLUT* glucose transporter; *AcCoA* acetyl-coenzyme A; *TCA* tricarboxylic acid cycle; *ANT* adenine nucleotide translocase; *CK* creatine kinase; *ETC* electron transport chain; *NADH* nicotinamide adenine dinucleotide; *FADH*_*2*_ flavin adenine dinucleotide; *Cr* creatine; *PCr* phosphocreatine; *CrT* creatine transporter

MRS is based on the same basic physical principles and uses similar equipment as MRI and can provide information on metabolites containing nuclei with a (net nonzero) nuclear spin. The most widely studied nuclei are phosphorus (^31^P), hydrogen (^1^H) and to a lesser extent sodium (^23^Na). The advent of hyperpolarisation techniques (DNP–dynamic nuclear polarisation) has opened a new field for carbon-13 (^13^C)-MRI/MRS. Each of these nuclei assesses different aspects of cardiac metabolism as illustrated in Fig. 1. ^31^P-MRS gives insight into high-energy phosphate metabolism (i.e., PCr, ATP and P_i_) and intracellular pH. ^23^Na can detect changes in intracellular and extracellular myocardial sodium content (ion homoeostasis). Due to the recent development of DNP, which increases the sensitivity by up to 5 orders of magnitude, ^13^C holds great potential to study multiple metabolic pathways such as glycolysis, tricarboxylic acid (TCA) cycle or β-oxidation in the myocardium. However, it is limited by the very short half-life time of the hyperpolarised nucleus and the need to inject supra-physiological concentrations of labelled substrate. Moreover, its clinical applicability and value for the characterisation of human hearts has yet to be demonstrated.

**Fig. 2:**
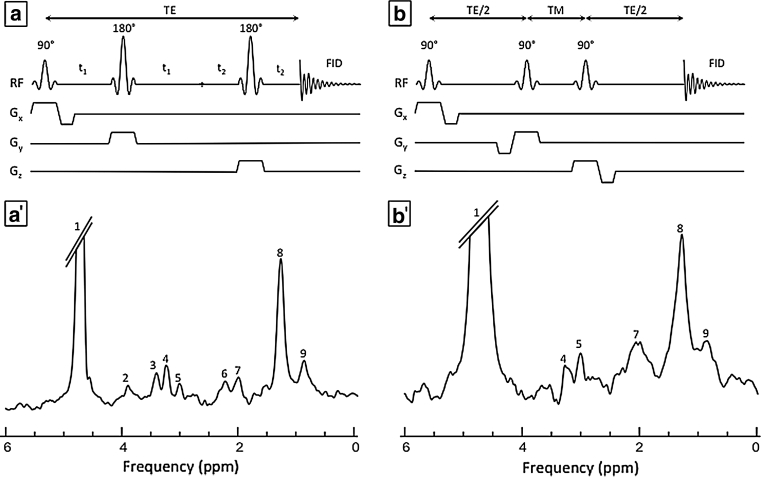
Schematic representation of single-voxel techniques that have been used to measure cardiac metabolites in the interventricular septum of mice (**a**, **a**′) and human (**b**, **b**′) hearts. **a** A cardiac- and respiratory-gated double spin-echo sequence (PRESS) was used to localise a 2 × 1 × 1 mm^3^ voxel in the myocardium of a C57BL/6J mouse (256 averages were applied). **b** A cardiac-gated stimulated echo sequence (STEAM) was applied 35 times over 5 breath-hold of approximately 16 s to acquire the signal from a 32 × 18 × 22 mm^3^ voxel in the human heart of an healthy volunteer. The main resonances are assigned as residual (1) water, 4.7 ppm; (2) creatine (–C**H**_**2**_), 3.88 ppm; (3) taurine, 3.39 ppm; (4) trimethyl-ammonium compounds, 3.21 ppm; (5) creatine (–C**H**_**3**_), 2.99 ppm; (6) triglycerides C_α_ **H**_2_COO, 2.21 ppm, (7) triglycerides (C**H**_**2**_–CH = CH–CH_2_), 1.99 ppm; (8) triglycerides (–CH_2_)_n_, 1.28 ppm; (9) triglycerides (CH_3_), 0.84 ppm. *TE* echo time; *TM* mixing time; *FID* free induction decay; *RF* radiofrequency pulses.—*Human spectrum (b′) courtesy of Dr B. Rial*

The proton (^1^H) is the nucleus with the highest NMR sensitivity and natural abundance in living tissue (>99.9 %). Therefore, ^1^H-MRS can potentially become a powerful technique as it can detect and quantify a variety of myocardial metabolites including lipids, taurine, carnitine and total creatine (Fig. 2). Lactate has also been detected in the hypoxic perfused rat heart [6, 7]. However, due to its low concentration and overlapping frequency range with the lipid resonance, its detection relies on excellent spectral resolution and/or the use of techniques to suppress the lipid signal. Finally, deoxy-/oxy-myoglobin resonances have been used to quantify myocardial oxygen availability ex vivo [8] or in vivo during high work states [9–11].

In vivo, ^1^H-MRS is mainly used to provide a window into cardiac metabolism at two different levels. Firstly, lipid (triglyceride) content of the heart is highly dynamic and myocardial lipid overload has been implicated in the pathophysiology of cardiac disease. Thus, measuring lipids will help to better understand their role in the healthy and diseased heart. Secondly, the measurement of total creatine, a crucial actor in the creatine kinase system, gives an insight into high-energy phosphate transport and storage. However, ^1^H-MRS is technically challenging and, up to now, necessitates specialist expertise and a dedicated set-up, which are the main reasons why the application of this otherwise versatile technique is still limited in clinical cardiology—despite considerable potential.

Clinical and preclinical studies are complementary for studying cardiac metabolism in the healthy and diseased heart. We will therefore initially provide a brief overview of methodological challenges and solutions, following which we will review the major findings in lipid and creatine metabolism in both patients and animal models using ^1^H-MRS. We will conclude with an outlook into future directions.

## Methodological considerations

Since the acquisition of the first ^1^H-MRS spectrum of an intact (perfused) rat heart in 1984 [12], it took 10 more years until the first human heart spectra were published [13]. Compared to the application of cardiac imaging, surprisingly, very little has since then been reported on the application of this technique in clinical and preclinical CMR. The main reason why ^1^H-MRS has not yet fulfilled its promise in clinical cardiology is related to its fundamental methodological challenges: (1) the metabolites studied with MRS are present in concentrations that are several orders of magnitude lower (typically in the mmol/l range) than those of water protons (present in concentrations of ~85 mol/l in tissue), resulting in larger voxel sizes and longer scan times compared to MRI as many signal averages are needed to obtain a sufficient signal-to-noise ratio (SNR). (2) The influence of cardiac and respiratory motion has to be minimised as this can negatively impact on the SNR. (3) The dominant water signal needs to be sufficiently suppressed to detect the weak metabolite signals. (4) Efficient water suppression and separation of the various metabolites in the spectrum require an excellent homogeneity of the static magnetic field, which is difficult to achieve especially in heterogeneous tissues (i.e., myocardium, blood, skeletal muscle, lungs) and is further hampered by the effect of motion. (5) The post-processing and data analysis is technically demanding and requires the consideration and correction of various experimental parameters if absolute metabolite concentrations are to be obtained. This list indicates that ^1^H-MRS is a technically complex method and requires a committed, interdisciplinary team of physicists, biochemists and clinical cardiologists to overcome these challenges. The technical requirements for a successful application of ^1^H-MRS in vivo are summarised below.

### Hardware and MR setup

Cardiac ^1^H-MRS studies use the same hardware (i.e., magnet, gradient and radiofrequency (RF) coils) as any cardiac MRI examination. A higher static magnetic field strength not only increases the signal-to-noise ratio of the experiment, but also benefits the spectral resolution. Human ^1^H-MRS studies have been possible on MR systems with a magnetic field strength of 1.5 and 3 Tesla [13–16], while we were the first to report on this technique in the mouse heart in vivo using an 11.7 Tesla magnet [17]. Preclinical CMR studies in small and large animal models have the additional complexity of anaesthesia and guaranteeing animal welfare during the MR examination.

Given the location of the heart inside the body, dedicated localisation techniques are required to obtain the metabolite signals from the myocardium only. The most commonly used single-voxel techniques, based on either a double spin-echo method (PRESS–Point-RESolved Spectroscopy sequence) [18] or stimulated echo sequence (STEAM–STimulated Echo Acquisition Mode sequence) [19], are schematically depicted in Fig. 2a, b respectively. Both require the application of three RF-pulses, each of which excites a plane orthogonal to each other. The resulting volume (i.e., voxel), from which the metabolite signals originate, is defined by the intersection of the three planes. More details about these pulse sequences can be found elsewhere [20, 21]. The voxel for cardiac MRS is typically placed in the inter-ventricular septum to avoid contamination of the spectrum with signals arising from pericardial fat. Metabolites in the blood have been shown not to contribute significantly to the final spectrum [14, 15, 17]. The MRS sequence is synchronised with the heartbeat, using the ECG signal. Multiple methods have been used to control for respiratory motion, including breath-holds, respiratory gating [22] or post-acquisition processing [23]. The main advantage of the breath-hold technique is that it does not require a complicated set-up [24]. While myocardial lipids can even be reproducibly measured within a single breath-hold [15], multiple breath-holds, if required, may become uncomfortable for patients and can lead to irreproducible position of the diaphragm [25]. Other motion-compensation techniques, including the use of a pressure belt registering the movements of the chest [16], the detection of the variation of the ECG amplitude during respiratory events [25] and, more recently, the application of navigator signals, which sense the displacement of the diaphragm [26, 27], have shown a good reproducibility.

Suppression of the dominant water signal is preferred even if modern analogue-to-digital converters (ADCs) provide a sufficient dynamic range to digitally resolve the weak metabolite and the large water signal simultaneously. However, the presence of a 3–5 order of magnitude stronger water resonance can create baseline distortions in the spectrum and render metabolite quantification unreliable, especially for metabolites with a chemical shift close to that of water (such as the methylene resonance of creatine). Water suppression schemes, such as chemical shift selective saturation [28], or saturation enhanced through T1 effects [29], are therefore typically applied prior to the spectroscopic sequence.

Depending on the spectral resolution and on the hardware used, a typical ^1^H-MRS study required between 15 and 30 min, which can potentially be a problem in unstable patients. In preclinical studies, a careful monitoring of cardiorespiratory function and body temperature is essential during the MRS-experiment.

### Post-processing and quantification

Unlike in the case of (cardiac) MR images, the information contained in an MR spectrum is less intuitive, except when there is an obvious deviation from the norm by the presence/absence of a resonance in the spectrum. Hence, computational fitting of the data either in the acquisition (i.e., time) domain or in its Fourier (i.e., frequency) domain is required to obtain a quantitative measure of a metabolite signal. This process is hampered not only by the low signal-to-noise in the data, but also by an imperfect model function used to approximate the data. Motion, magnetic field inhomogeneities, residual eddy currents (caused by fast switching magnetic field gradients required for water suppression and/or localisation) and a rolling baseline in the spectra (resulting from the residual water) may place additional constraints on the quantitative analysis of ^1^H-MR spectra and may make robust fitting very difficult. Frequency- and phase correction of each acquisition prior to averaging, based on either the residual water signal or the lipid signal, have been shown to improve at least the line-shape and the SNR.

Absolute quantification, that is, the measurement of metabolite concentrations in mmol/l requires the use of a concentration standard (external or internal), and the correction of all parameters intervening in absolute quantification, such as the influence of relaxation/saturation, RF-pulse inhomogeneity, RF-coil sensitivity etc. It is therefore more common to acquire one water-suppressed and one water-unsuppressed spectrum from the same location in the myocardium and to quantify the metabolites relative to the water content as an internal reference. This approach compensates for any hardware influence, such as RF-profile or eddy currents, but will fail if the tissue water content is altered. Most of the applications reported in the following two sections have used this semi-quantitative approach.

## Lipid metabolism

Since the first detection of myocardial lipids in vivo in the human heart [13] in 1994, ^1^H-MRS has been shown to be a reliable and reproducible technique to quantify lipid content. As illustrated in Fig. 2, multiple lipid resonances can be observed in a typical cardiac ^1^H-spectrum, which are attributable mostly to the methyl and methylene resonances of triglycerides, although contribution from other lipids such as cellular phospholipids cannot be excluded [30]. The methylene resonance at 1.28 ppm is commonly used for quantification due to the higher concentration of methylene protons in fat and referenced to the (unsuppressed) water signal from the same region of interest. Validation on animal hearts showed a good correlation between spectroscopic and standard biochemical measurements [16]. Moreover, the technique is ideally suited for longitudinal studies due to its high long-term reproducibility rate; a coefficient of variation of only 5 % was observed by Reingold et al. [31] over a 90-day period. The myocardial lipid pool is highly dynamic, and in physiological conditions, the myocardial triglyceride (Tg) content can vary significantly. Therefore, it is important to understand the possible sources of physiological variations in the normal heart before describing the role of abnormal lipid levels in the disease state. We will focus on the most common pathologies affecting the heart, such as obesity and type 2 diabetes mellitus, myocardial infarction, cardiomyopathy and heart failure (HF).

### Physiological variations of Tg in the healthy heart

In healthy non-obese individuals, the cardiac lipid content in the fasted state is relatively consistent between studies with an average lipid/water ratio ranging from 0.3 to 0.6 % [15, 31–34]. However, multiple physiological factors may influence the abundance of this lipid pool, and it is crucial to understand and acknowledge these sources of variation as they may also impact on the quantifiable lipid levels in cardiac disease.

The lipid content primarily depends on the feeding state. In two independent studies, myocardial lipid content was increased by 3- to fourfold, following a fasting period of 2 and 3 days, respectively [31, 35], whereas it increased by only 55–70 % in the case of partial caloric restriction [35, 36]. This was associated with increases in plasma FFA proportional to the degree of starvation [35]. Interestingly, these changes in lipid metabolism are paralleled by an impairment of diastolic function, correlating with the increase in myocardial Tg content [35, 36].

Prolonged exercise also resulted in an increased Tg storage in cardiac tissue (accompanied by a rise in plasma FA levels), but this did not hamper systolic cardiac function as assessed by cine-MRI [34]. Conversely, 3 days on a high-fat/high-energy diet did not induce any detectable change in myocardial Tg content despite the significant increases in post-prandial plasma triglyceride, FFA and hepatic triglyceride content [37]. Differences in metabolic state could explain why increased plasma FFA levels are associated with an increase in myocardial Tg content in the case of caloric restriction and not during caloric excess [37].

**Fig. 3:**
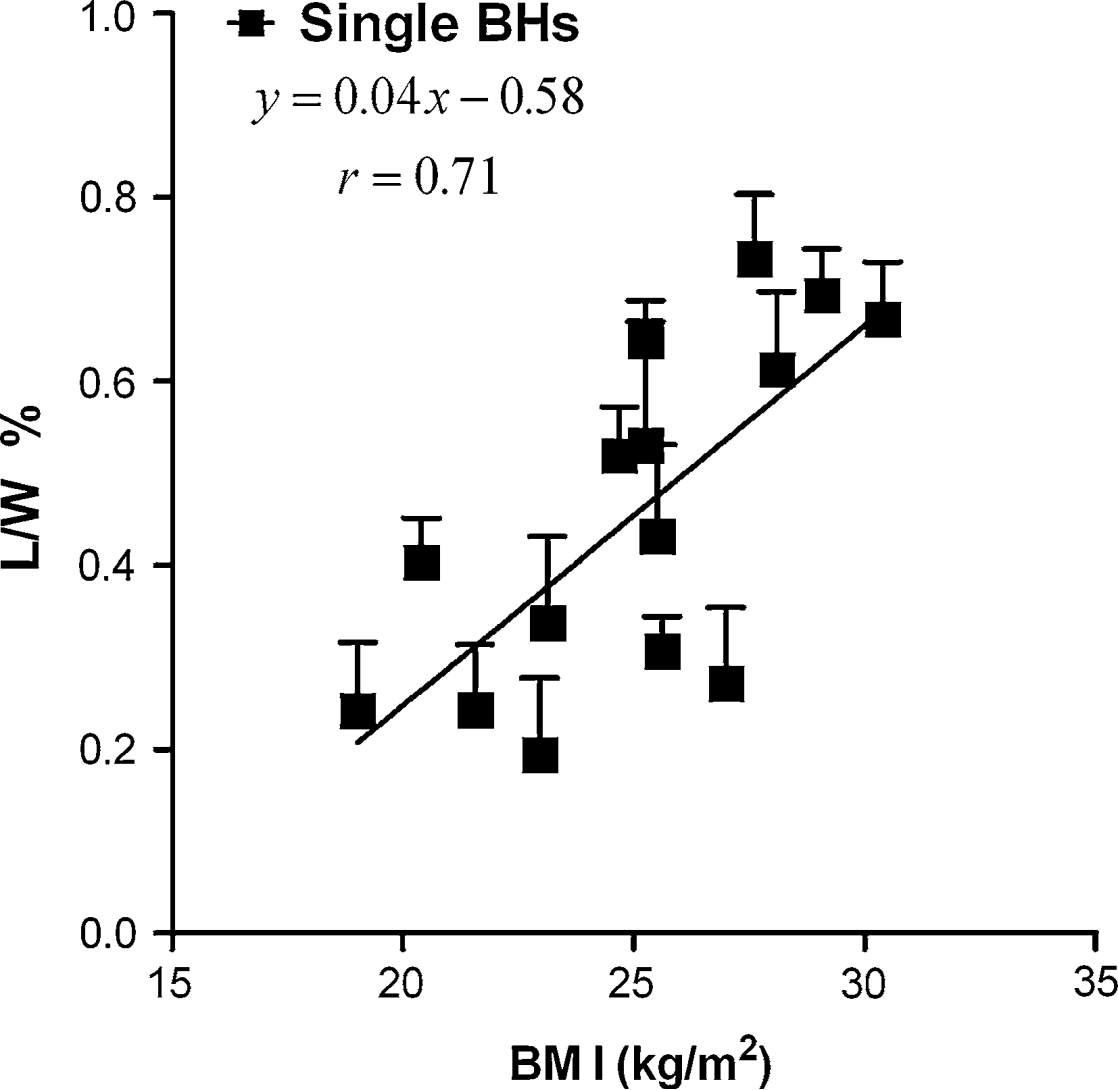
Correlation between myocardial lipid levels (L/W%) assessed by ^1^H-MRS and BMI in healthy volunteers. Signal was acquired within a single breath-hold from a 22 × 12–19 × 32–36 mm voxel located in the interventricular septum using a STEAM pulse sequence. (From [15]—reproduced with kind permission from John Wiley and Sons)

The main determinant of myocardial Tg content is the body mass index, and a good correlation between BMI and Tg over a wide range of BMI has been demonstrated (Fig. 3) [15, 38]. However, other factors may also contribute to individual variations. Specifically, van der Meer et al. [32] showed an increase in the Tg content in the ageing heart, associated with the age-related decline in diastolic function, which was independent of the BMI. However, this finding was not confirmed in a different study [38].

### Obesity and type 2 diabetes mellitus

Obesity and its associated comorbidities, which include type 2 diabetes mellitus, are reaching pandemic proportion [39, 40]. They are referred to as the metabolic syndrome and could result in a decrease in the life expectancy in the 21st century [41, 42]. Obesity (i.e., body mass index (BMI) ≥ 30 kg/m^2^), which is the consequence of an imbalance between caloric intake and energy expenditure, leads to triglyceride storage in organs other than adipose tissue, such as muscle, liver, pancreas and also the heart. The ectopic deposition of these lipid droplets in the cytoplasm results in cell dysfunction, a phenomenon called lipotoxicity [42, 43].

Despite this concept of fatty heart having been described for the first time more than 300 years ago, its implications on cardiac function were only appraised in the second half of the 20th century with the development of echocardiography [44, 45]. Alpert et al. [45] reported a correlation between the increase in LV mass, decrease in systolic function and impairment in diastolic filling observed in morbidly obese patients and the duration of obesity. Moreover, the large-scale Framingham study showed a significant link between LV mass and BMI even after adjustment for potential confounding factors such as age or blood pressure [46–48].

In an animal model of obesity secondary to unresponsiveness to leptin, the ZDF rat, myocardial steatosis has been associated with cardiac dysfunction secondary to lipoapoptosis [48]. This fatty acid infiltration has also commonly been observed in the non-ischaemic failing heart of patients at the time of transplantation, particularly in obese and diabetic people. Interestingly, this metabolic dysregulation was accompanied with a distinct transcriptional profile similar to the one observed in the ZDF hearts [49].

CMR studies in obese patients can be hampered by significantly reduced patient comfort due to the restricted diameter of the magnet bore (typically in the order of 60 cm), which may result in a more frequent occurrence of claustrophobia. Reduced sensitivity due to the increased distance between receive coil-array and the heart, breathing problems, affecting breath-hold, resulting in variable breath-hold times, and/or ECG detachment due to sweating may place additional constraints on the application of ^1^H-MRS in this patient group. Nevertheless, ^1^H-MRS studies have been feasible and have confirmed an increase in myocardial lipid content in hearts of overweight and obese subjects by up to threefold compared to lean individuals [15, 16, 24]. This triglyceride overload was associated with an increase in cardiac mass and a subtle impairment in myocardial systolic thickening [16]. Following dietary restriction in obese diabetic patients, myocardial triglyceride content decreases, which was associated with an improvement in diastolic function (E/A ratio) [35]. Improvement in cardiac function also paralleled the decrease in cardiac Tg, following either a therapeutic intervention (administration of trimetazidine [50, 51]) or exercise [51].

The existence of a diabetic cardiomyopathy has been recognised since 1972 [52]. While myocardial fatty acid accumulation and the associated lipotoxicity seem to play a role, the exact underlying aetiology is still incompletely understood [53]. In a large human study using ^1^H-MRS, McGavock et al. [33] showed that cardiac steatosis occurs in patients with impaired glucose tolerance even before the onset of type 2 diabetes mellitus and precedes cardiac dysfunction. Another study compared patients with type 2 diabetes mellitus (T2DM) with controls of the same age and BMI and showed that there is an increase in myocardial triglyceride content in uncomplicated T2DM and is associated with an impairment of LV diastolic function [54]. These studies constitute the first step towards a better understanding of diabetic cardiomyopathy in the human heart, and, as stated by van de Weijer et al. [53], the development of non-invasive techniques to assess cardiac metabolism should bring an important contribution to this field.

^1^H-MRS to assess cardiac lipid metabolism in pre-clinical studies is still under-utilised despite its potential. Bakermans et al. [55] have successfully applied the technique in transgenic mice deficient in long-chain acyl-CoA dehydrogenase. These mice were characterised by myocardial Tg accumulation (normal: 0.65 ± 0.16 % of water signal; transgenic: 1.10 ± 0.20 %), which further increased by 63 % upon fasting. Concomitantly, an impairment in systolic and diastolic function was observed in the fasted animals suggesting toxicity associated with accumulating lipid metabolites.

### Myocardial infarction

Changes in fatty acid metabolism occur following myocardial infarction (i.e., temporary or permanent interruption of the coronary blood supply). In the central zone of the infarct, the drastic decrease in blood flow quickly leads to cell death. However, in the border zone where blood flow decreases less severely, cellular integrity is conserved and a reduction in β-oxidation results in the accumulation of lipid droplets within myocardial cells [56, 57]. Changes in lipid content in this area-at-risk can be assessed by magnetic resonance.

In canine hearts following 24-h coronary occlusion, lipid content—measured ex vivo by high resolution ^1^H-NMR—was increased in areas with moderate blood flow reduction compared to control areas or areas with severe obstruction [58]. Follow-up studies on the same model were performed on the intact excised heart using cardiac spectroscopic imaging and confirmed that triglycerides accumulate in the area-at-risk, resulting in an increase by up to 10 times compared to the remote zone [59, 60]. Finally, investigating the “stunned” canine myocardium induced by 15 min of coronary occlusion followed by 3 h of reperfusion also showed a twofold increase in NMR detectable lipid content in the injured area [61].

Therefore, lipid accumulation may potentially represent a clinical tool to assess the area-at-risk. However, this requires excellent spatial localisation in order to reliably assess cardiac lipids in a given cardiac area, which is particularly problematic for the free wall due to the potential for signal contamination from the adjacent lipid-rich pericardium.

### Cardiomyopathy and heart failure

To date, only very few studies comprising small numbers of patients have focused on lipid metabolism in cardiomyopathy and heart failure. In a study on hypertrophic cardiomyopathy (HCM), dilated cardiomyopathy (DCM) and ischaemic cardiomyopathy, Nakae et al. [38] demonstrated that there is no consistent effect of the size of the Tg pool with cardiac dysfunction. Lipid concentration was decreased in HCM and correlated with left ventricular ejection fraction (LVEF), while there was a very wide variation in DCM. In the latter, Tg content did not correlate with cardiac dysfunction, but significantly with BMI. These findings agree with the observations by Sharma et al. [49], who found that, in the non-ischaemic cardiac HF model, accumulation of lipids was most pronounced when heart failure was associated with diabetes and obesity.

## Creatine metabolism

In tissues with high and fluctuating energy demand such as the heart, the creatine kinase (CK) system plays a crucial role in the transport and buffering of high-energy phosphates from sites of production (i.e., mitochondria) to sites of utilisation (i.e., myofibrils). CK catalyses the reversible transfer of the γ-phosphate group of ATP to creatine yielding adenosine diphosphate (ADP) and phosphocreatine. The latter serves as an energy carrier and reserve, replenishing ATP levels and maintaining low ADP levels in the vicinity of cellular ATPases.

Changes in high-energy phosphate metabolism occur in the failing heart of humans and animals: a decrease in phosphocreatine and total creatine by up to ~60 % independent of the underlying aetiology has been measured in numerous studies and precedes the drop in ATP observed in end-stage HF. These observations have been at the origin of the energy-starvation hypothesis of the failing heart [62]. However, the exact role of creatine in the pathophysiology of HF is still unclear, and determining this has so far been hampered by a lack of sensitive, non-invasive techniques to assess high-energy phosphate metabolism. The development of MRS addresses this need, providing a window into cardiac metabolism that has sparked renewed interest in this field of cardiac research.

To date, more papers have reported on cardiac ^31^P-MRS in vivo, while only a handful of studies used ^1^H-MRS. The ratio of PCr/ATP is considered a sensitive indicator of the metabolic state of the myocardium, as it decreases in the hypertrophied and failing human and animal heart [63–65]. Interestingly, it has also been shown to be a better prognostic marker in patients with dilated cardiomyopathy than the traditionally used New York Heart Association class or LV ejection fraction [66]. However, this ratio remains a surrogate of absolute measurement in the absence of a widely available method to non-invasively quantify PCr and ATP concentrations. In the presence of reduced ATP levels, this factor will underestimate the true extent of high-energy phosphate depletion. Moreover, in the presence of heterogeneous cardiac disease, regional variations have to be considered. Specifically, it has recently been shown in a swine model of MI using 2D-CSI that no ATP or PCr could be detected in the infarcted tissue, whereas PCr/ATP was severely reduced in the border zone compared to the remote zone [67].

^1^H-MRS allows the assessment of the total creatine pool (sum of the free creatine and PCr). Hence, ^31^P-MRS and ^1^H-MRS provide complementary information, and, by combining both techniques, a near complete profile of the CK system can be obtained: ^31^P-MRS allows for direct measurement of ATP & PCr levels, and ^1^H-MRS can quantify total creatine [68], which enables the calculation of ADP levels from the CK reaction equilibrium:

1 $$ \left[ {\text{ADP}} \right] = \left( {\left[ {\text{ATP}} \right] \times \left[ {\text{Free Cr}} \right]} \right)/\left( {\left[ {\text{PCr}} \right] \times \left[ {{\text{H}}^{ + } } \right] \times K_{\text{eq}} } \right), $$

where *K*_eq_ is the equilibrium constant of the reaction, and [ADP], [ATP], [Free Cr], [PCr], [H^+^] indicate the concentrations in ADP, ATP, free creatine, phosphocreatine and protons, respectively.

This approach represents, in principle, a unique opportunity to non-invasively measure in vivo the free energy change of ATP hydrolysis (Δ*G*_~ATP_) available to drive ATP-requiring reactions, according to the equation:

2 $$ \Updelta G_{{\sim {\text{ATP}}}} \left( {\text{kJ/mol}} \right) = \Updelta G^\circ + RT{ \ln }\left( {\left[ {\text{ADP}} \right] \times \left[ {\text{Pi}} \right]/\left[ {\text{ATP}} \right]} \right), $$

where Δ*G*° is the value of Δ*G*_~ATP_ under standard conditions of molarity, temperature, pH and Mg^2+^; *R* the universal gas constant; *T*, the temperature (in Kelvin); and [Pi], the concentration in inorganic phosphate.

Maintaining a high |Δ*G*_~ATP_| is essential for normal myocyte function. Small changes in the free energy of ATP hydrolysis would impede the activity of key intracellular ATPases such as the sarco/endoplasmic reticulum Ca^2+^-ATPase, the myosin ATPase or the Na^+^-K^+^-ATPase. Ingwall and Shen [69] called *energy reserve* the difference between the energy available from ATP hydrolysis and the driving force required for the ATP-utilising reactions to occur. In the failing heart or at high workloads, this energy reserve might become critical for the reaction to occur. Therefore, augmenting the high-energy phosphate metabolism could be an effective therapeutic strategy to support the energy-starved failing heart, as recently demonstrated by Gupta et al. They showed that increasing ATP flux by overexpressing the myofibrillar isoform of CK in mice, improved contractile function and long-term survival following thoracic aortic constriction [5].

### Acute myocardial ischaemia

Bottomley and Weiss [14] were first to use ^1^H-MRS to measure total creatine in the human heart. After validation of the technique in a canine model of myocardial infarction [68], they measured in patients approximately threefold decrease in creatine levels in the infarcted area compared to the remote myocardium or healthy control [14, 68]. The potential of ^1^H-MRS in assessing ischaemic myocardium metabolism was also demonstrated by a study investigating myocardial contractility. In patients with a stenosis of the left anterior descending coronary artery of 50 % or more, Nakae et al. [70] showed a decrease in total creatine levels by about 40–50 % in the hypokinetic and akinetic regions of the ischaemic myocardium, whereas in patients with no wall motion abnormalities, creatine levels were comparable to those measured in healthy controls. These results confirm ex vivo NMR observations on tissue samples from the infarcted canine heart, where creatine levels decreased in proportion to the level of coronary flow reduction [58] and suggest that ^1^H-MRS could be a suitable technique in appraising myocardial viability in coronary artery disease.

### Chronic heart failure

**Fig. 4:**
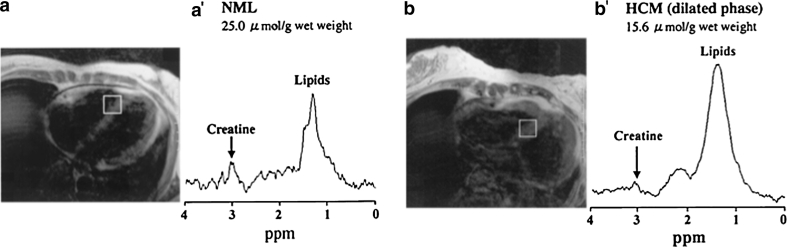
**a**′ shows a spectrum acquired from the interventricular septum of a normal healthy volunteer (**a**). **b**′ shows a spectrum acquired from the same location in a patient with advanced hypertrophic cardiomyopathy (dilated phase). A decrease in the creatine peak amplitude can be noted. (From [71]—reproduced with kind permission from Elsevier)

In a series of ^1^H-MRS studies, Nakae et al. [38, 71–73] have shown a significant reduction in myocardial creatine content in patients with heart failure secondary to non-ischaemic diseases (dilated cardiomyopathy, hypertrophic cardiomyopathy, cardiac amyloidosis or valvular heart disease). All these studies were limited by the small number of patients enrolled, which prevented a statistically powerful individual analysis of each disease. However, the importance of creatine depletion seemed to reflect the severity of heart failure, and this was found to be independent of the aetiology of HF (Fig. 4). Myocardial creatine concentration correlated positively with cardiac function (LVEF) [38, 71, 73] and free fatty acid uptake [38] and negatively with plasma brain natriuretic peptides (BNP) levels [71, 72]. Finally, total creatine levels could potentially be predictive of subsequent heart failure; Nakae et al. [73] showed that creatine levels had been lower in patients who were later admitted to hospital for heart failure in the year following the metabolic assessment (11.3 ± 1.0 vs. 18.6 ± 5.9 µmol/l). However, these findings need to be confirmed prospectively in larger studies and compared with standard predictive markers. It also still remains to be clarified whether this decrease in creatine level contributes to the development of heart failure or could constitute an adaptive process to maintain the ATP/ADP ratio, thus preserving the chemical driving force of ATPases [74, 75].

### Understanding the role of creatine with transgenic models

The exact role of creatine depletion and more broadly of energy depletion in the pathophysiology of HF is still unclear. Pharmaceutical inhibition and more recently transgenic mouse models have provided a new opportunity to study the specific roles of the different components of the CK system. They also constitute an invaluable tool for testing the hypothesis that enhancing (or preventing the decline of) energy availability would improve cardiac function in the failing heart.

**Fig. 5:**
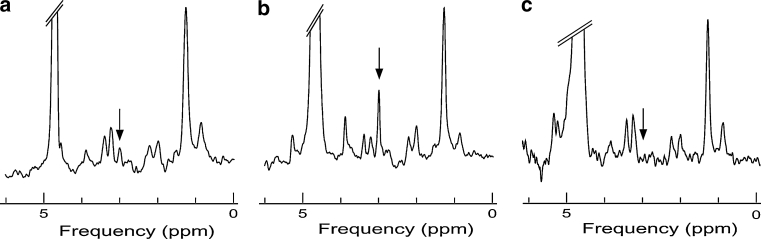
^1^H-MRS spectra from the hearts of wild-type (**a**), creatine transporter over-expressor (CrT-OE) (**b**) and guanidinoacetate *N*-methyltransferase knock-out (GAMT-ko) (**c**) mice. The *arrows* show the methyl resonance of creatine. Note the very high creatine peak in the cardiac spectrum of the creatine transporter over-expressor mouse compared to other resonances (**b**) and the lack of creatine resonance in the GAMT-ko mouse (**c**)

To address this question, we sought to increase myocardial creatine content. This cannot be achieved by dietary creatine supplementation due to negative-feedback regulation of creatine transporter activity, which provides tight regulation of creatine levels in the heart [76]. To bypass this mechanism, we have generated a transgenic mouse model over-expressing the myocardial creatine transporter (CrT-OE mice) [77], whose downregulation likely explain the decrease in creatine content in the diseased myocardium [78]. Using ^1^H-MRS and cine-MRI, creatine metabolism and cardiac function were monitored over 1 year in these transgenic mice. We found a large variation in creatine elevation between transgenic mice, which then remained constant throughout an individual’s lifetime. To our surprise, very high levels of myocardial creatine (Fig. 5b) were associated with a progressive decline in cardiac function during ageing, most likely due to reduced glycolytic activity [79] and an inability to keep the total creatine pool adequately phosphorylated [77]. However, it remains to be tested whether a more modest increase in intracellular creatine may yet be beneficial in chronic heart failure. In an ongoing study, we are using ^1^H-MRS to prospectively select CrT-OE mice with known creatine levels prior to experimental myocardial infarction, an approach that would not be possible using any other technology.

Another approach is to study the hearts of mice with no creatine. In a transgenic model (GAMT-KO) unable to biosynthesise creatine, we confirmed non-invasively the absence of creatine in the myocardium of these mice (Fig. 5c) [17]. While the creatine-free GAMT-KO mice had a mild cardiac phenotype under baseline conditions (most likely due to compensatory mechanisms), they exhibited a reduction in inotropic reserve and an enhanced susceptibility to ischaemia/reperfusion [80]. Other experimental designs become available by utilising longitudinal ^1^H-MRS and creatine-deficient mice, such as withdrawal of dietary creatine to determine kinetics of myocardial creatine loss and creatine supplementation to determine whether the phenotype can be rescued.

## Conclusion and future directions

Over the last decade, ^1^H-MRS has become an invaluable technique to non-invasively assess cardiac lipid and to a lesser extend creatine metabolism in clinical and pre-clinical research; major findings are summarised in Table 1. However, technical challenges, limited spatial and temporal resolution have hampered its widespread development, especially as a clinical diagnostic tool. Both the use of higher field strength magnets and advances in RF-coil design and the development of more sophisticated motion-compensation schemes (i.e., navigator techniques) combined with advanced MRS-pulse sequences should significantly improve the robustness of the technique and allow for shorter acquisition times [81]. This is a necessary condition for proton (and other nuclei) spectroscopy to become, in the future, an integral part of a standard CMR protocol. Examination of regionally heterogeneous cardiac pathologies such as ischaemic heart disease would also clearly benefit from further development of magnetic resonance spectroscopic imaging techniques. Finally, for MRS to expand beyond research, improvements in quantitative analysis of MR spectra are required, to provide instantaneous robust results that can be interpreted by the cardiologist. With these future developments in sight, MRS will not only be established as an invaluable research tool for the study of myocardial metabolism in animal models of cardiovascular disease, but may also become more widely used in clinical practice.

**Table 1:** Summary of the major sources of physiological variation of myocardial lipid and creatine levels

	Lipids	Creatine
Starvation	⇑ (correlates to degree of starvation)	NA
Obesity	⇑ (correlates to BMI)	NA
Diabetes mellitus type 2	⇑	NA
Infarction	⇑ in area-at-risk	⇓⇓ in infarcted area
Hypertrophic cardiomyopathy	⇓ shown in one study	⇓ proportional to cardiac dysfunction
Dilated cardiomyopathy	No clear pattern	⇓ proportional to cardiac dysfunction

## References

[1] The NHS Information Centre for Health and Social Care 2010 (2011) National heart failure audit 2010 [online] http://www.ic.nhs.uk/webfiles/publications/002_Audits/NHS_IC_National_Heart_Failure_Audit_2010_04-01-11.pdf

[2] Neubauer S (2007). The failing heart–an engine out of fuel. N Engl J Med.

[3] Opie LH, Lopaschuk GD, Opie LH (2004). Fuels: aerobic and anaerobic metabolism. Heart physiology: from cell to circulation.

[4] Taegtmeyer H (1994). Energy metabolism of the heart: from basic concepts to clinical applications. Curr Probl Cardiol.

[5] Gupta A, Akki A, Wang Y, Leppo MK, Chacko VP, Foster DB, Caceres V, Shi S, Kirk JA, Su J, Lai S, Paolocci N, Steenbergen C, Gerstenblith G, Weiss RG (2012). Creatine kinase-mediated improvement of function in failing mouse hearts provides causal evidence the failing heart is energy starved. J Clin Invest.

[6] Keller AM, Sorce DJ, Sciacca RR, Barr ML, Cannon PJ (1988). Very rapid lactate measurement in ischemic perfused hearts using 1H MRS continuous negative echo acquisition during steady-state frequency selective excitation. Magn Reson Med.

[7] Schussheim AE, Radda GK (1993). Study of ischemic myocardial buffering capacity in perfused rat heart through rapidly interleaved 1H and 31P MRS measurements. Magn Reson Med.

[8] Kreutzer U, Jue T (1991). 1H-nuclear magnetic resonance deoxymyoglobin signal as indicator of intracellular oxygenation in myocardium. Am J Physiol.

[9] Kreutzer U, Mekhamer Y, Chung Y, Jue T (2001). Oxygen supply and oxidative phosphorylation limitation in rat myocardium in situ. Am J Physiol Heart Circ Physiol.

[10] Murakami Y, Zhang Y, Cho YK, Mansoor AM, Chung JK, Chu C, Francis G, Ugurbil K, Bache RJ, From AH, Jerosch-Herold M, Wilke N, Zhang J (1999). Myocardial oxygenation during high work states in hearts with postinfarction remodeling. Circulation.

[11] Zhang J, Murakami Y, Zhang Y, Cho YK, Ye Y, Gong G, Bache RJ, Ugurbil K, From AH (1999). Oxygen delivery does not limit cardiac performance during high work states. Am J Physiol.

[12] Ugurbil K, Petein M, Maidan R, Michurski S, Cohn JN, From AH (1984). High resolution proton NMR studies of perfused rat hearts. FEBS Lett.

[13] den Hollander JA, Evanochko WT, Pohost GM (1994). Observation of cardiac lipids in humans by localized 1H magnetic resonance spectroscopic imaging. Magn Reson Med.

[14] Bottomley PA, Weiss RG (1998). Non-invasive magnetic-resonance detection of creatine depletion in non-viable infarcted myocardium. Lancet.

[15] Rial B, Robson MD, Neubauer S, Schneider JE (2011). Rapid quantification of myocardial lipid content in humans using single breath-hold 1H MRS at 3 Tesla. Magn Reson Med.

[16] Szczepaniak LS, Dobbins RL, Metzger GJ, Sartoni-D’Ambrosia G, Arbique D, Vongpatanasin W, Unger R, Victor RG (2003). Myocardial triglycerides and systolic function in humans: in vivo evaluation by localized proton spectroscopy and cardiac imaging. Magn Reson Med.

[17] Schneider JE, Tyler DJ, ten Hove M, Sang AE, Cassidy PJ, Fischer A, Wallis J, Sebag-Montefiore LM, Watkins H, Isbrandt D, Clarke K, Neubauer S (2004). In vivo cardiac 1H-MRS in the mouse. Magn Reson Med.

[18] Ordidge RJ, Bendall MR, Gordon RE, Connelly A, Govil G, Khetrapal CL, Saran A (1985). Volume selection in vivo spectroscopy. Magnetic resonance in biology and medicine.

[19] Frahm J, Merboldt KD, Hanicke W (1987). Localized spectroscopy using stimulated echoes. J Magn Reson.

[20] Gadian DG, Gadian DG (1996). Pulse sequences. NMR and its application to living systems.

[21] de Graaf RA, De Graaf RA (2007). Single volume localization and water suppression. In vivo NMR spectroscopy: principles and techniques.

[22] Cassidy PJ, Schneider JE, Grieve SM, Lygate C, Neubauer S, Clarke K (2004). Assessment of motion gating strategies for mouse magnetic resonance at high magnetic fields. J Magn Reson Imaging.

[23] Gabr RE, Sathyanarayana S, Schär M, Weiss RG, Bottomley PA (2006). On restoring motion-induced signal loss in single-voxel magnetic resonance spectra. Magn Reson Med.

[24] Kankaanpää M, Lehto H-R, Pärkkä JP, Komu M, Viljanen A, Ferrannini E, Knuuti J, Nuutila P, Parkkola R, Iozzo P (2006). Myocardial triglyceride content and epicardial fat mass in human obesity: relationship to left ventricular function and serum free fatty acid levels. J Clin Endocrinol Metab.

[25] Felblinger J, Jung B, Slotboom J, Boesch C, Kreis R (1999). Methods and reproducibility of cardiac/respiratory double-triggered (1)H-MR spectroscopy of the human heart. Magn Reson Med.

[26] Schär M, Kozerke S, Boesiger P (2004). Navigator gating and volume tracking for double-triggered cardiac proton spectroscopy at 3 Tesla. Magn Reson Med.

[27] van der Meer RW, Doornbos J, Kozerke S, Schär M, Bax JJ, Hammer S, Smit JWA, Romijn JA, Diamant M, Rijzewijk LJ, de Roos A, Lamb HJ (2007). Metabolic imaging of myocardial triglyceride content: reproducibility of 1H MR spectroscopy with respiratory navigator gating in volunteers. Radiology.

[28] Haase A, Frahm J, Hanicke W, Matthaei D (1985). 1H NMR chemical shift selective (CHESS) imaging. Phys Med Biol.

[29] Ogg R, Kingsley P, Taylor J (1994). WET, a T1- and B1-insensitive water-suppression method for in vivo localized 1H NMR spectroscopy. J Magn Reson B.

[30] Zhou D, Guo Z (2012) Intramyocellular lipids versus intramyocellular triglycerides. Magn Reson Med 67:297–298 (author reply 299) 10.1002/mrm.23255 PMC4235960 22180024

[31] Reingold JS, McGavock JM, Kaka S, Tillery T, Victor RG, Szczepaniak LS (2005). Determination of triglyceride in the human myocardium by magnetic resonance spectroscopy: reproducibility and sensitivity of the method. Am J Physiol Endocrinol Metab.

[32] van der Meer RW, Rijzewijk LJ, Diamant M, Hammer S, Schär M, Bax JJ, Smit JWA, Romijn JA, de Roos A, Lamb HJ (2008). The ageing male heart: myocardial triglyceride content as independent predictor of diastolic function. Eur Heart J.

[33] McGavock JM, Lingvay I, Zib I, Tillery T, Salas N, Unger R, Levine BD, Raskin P, Victor RG, Szczepaniak LS (2007). Cardiac steatosis in diabetes mellitus: a 1H-magnetic resonance spectroscopy study. Circulation.

[34] Bilet L, Van De Weijer T, Hesselink MKC, Glatz JFC, Lamb HJ, Wildberger J, Kooi ME, Schrauwen P, Schrauwen-Hinderling VB (2011). Exercise-induced modulation of cardiac lipid content in healthy lean young men. Basic Res Cardiol.

[35] Hammer S, van der Meer RW, Lamb HJ, Schär M, de Roos A, Smit JWA, Romijn JA (2008). Progressive caloric restriction induces dose-dependent changes in myocardial triglyceride content and diastolic function in healthy men. J Clin Endocrinol Metab.

[36] van der Meer RW, Hammer S, Smit JWA, Frölich M, Bax JJ, Diamant M, Rijzewijk LJ, de Roos A, Romijn JA, Lamb HJ (2007). Short-term caloric restriction induces accumulation of myocardial triglycerides and decreases left ventricular diastolic function in healthy subjects. Diabetes.

[37] van der Meer RW, Hammer S, Lamb HJ, Frölich M, Diamant M, Rijzewijk LJ, de Roos A, Romijn JA, Smit JWA (2008). Effects of short-term high-fat, high-energy diet on hepatic and myocardial triglyceride content in healthy men. J Clin Endocrinol Metab.

[38] Nakae I, Mitsunami K, Yoshino T, Omura T, Tsutamoto T, Matsumoto T, Morikawa S, Inubushi T, Horie M (2010). Clinical features of myocardial triglyceride in different types of cardiomyopathy assessed by proton magnetic resonance spectroscopy: comparison with myocardial creatine. J Card Fail.

[39] Grundy SM (2008). Metabolic syndrome pandemic. Arterioscler Thromb Vasc Biol.

[40] Unger RH, Clark GO, Scherer PE, Orci L (2010). Lipid homeostasis, lipotoxicity and the metabolic syndrome. Biochim Biophys Acta.

[41] Olshansky SJ, Passaro DJ, Hershow RC, Layden J, Carnes BA, Brody J, Hayflick L, Butler RN, Allison DB, Ludwig DS (2005). A potential decline in life expectancy in the United States in the 21st century. N Engl J Med.

[42] van Herpen NA, Schrauwen-Hinderling VB (2008). Lipid accumulation in non-adipose tissue and lipotoxicity. Physiol Behav.

[43] Wende AR, Abel ED (2010). Lipotoxicity in the heart. Biochim Biophys Acta.

[44] Szczepaniak LS, Victor RG, Orci L, Unger RH (2007). Forgotten but not gone: the rediscovery of fatty heart, the most common unrecognized disease in America. Circ Res.

[45] Alpert MA, Lambert CR, Panayiotou H, Terry BE, Cohen MV, Massey CV, Hashimi MW, Mukerji V (1995). Relation of duration of morbid obesity to left ventricular mass, systolic function, and diastolic filling, and effect of weight loss. Am J Cardiol.

[46] Lauer MS, Anderson KM, Kannel WB, Levy D (1991). The impact of obesity on left ventricular mass and geometry. The Framingham Heart Study. JAMA.

[47] Kenchaiah S, Evans JC, Levy D, Wilson PWF, Benjamin EJ, Larson MG, Kannel WB, Vasan RS (2002). Obesity and the risk of heart failure. N Engl J Med.

[48] Zhou YT, Grayburn P, Karim A, Shimabukuro M, Higa M, Baetens D, Orci L, Unger RH (2000). Lipotoxic heart disease in obese rats: implications for human obesity. Proc Natl Acad Sci USA.

[49] Sharma S, Adrogue JV, Golfman L, Uray I, Lemm J, Youker K, Noon GP, Frazier OH, Taegtmeyer H (2004). Intramyocardial lipid accumulation in the failing human heart resembles the lipotoxic rat heart. FASEB J.

[50] Bucci M, Borra R, Någren K, Pärkkä JP, Del Ry S, Maggio R, Tuunanen H, Viljanen T, Cabiati M, Rigazio S, Taittonen M, Pagotto U, Parkkola R, Opie LH, Nuutila P, Knuuti J, Iozzo P (2011) Trimetazidine reduces endogenous free fatty acid oxidation and improves myocardial efficiency in obese humans. Cardiovasc Ther. doi:10.1111/j.1755-5922.2011.00275.x 10.1111/j.1755-5922.2011.00275.x 21884010

[51] Schrauwen-Hinderling VB, Hesselink MKC, Meex R, van der Made S, Schär M, Lamb H, Wildberger JE, Glatz J, Snoep G, Kooi ME, Schrauwen P (2010). Improved ejection fraction after exercise training in obesity is accompanied by reduced cardiac lipid content. J Clin Endocrinol Metab.

[52] Rubler S, Dlugash J, Yuceoglu YZ, Kumral T, Branwood AW, Grishman A (1972). New type of cardiomyopathy associated with diabetic glomerulosclerosis. Am J Cardiol.

[53] van de Weijer T, Schrauwen-Hinderling VB, Schrauwen P (2011). Lipotoxicity in type 2 diabetic cardiomyopathy. Cardiovasc Res.

[54] Rijzewijk LJ, van der Meer RW, Smit JWA, Diamant M, Bax JJ, Hammer S, Romijn JA, de Roos A, Lamb HJ (2008). Myocardial steatosis is an independent predictor of diastolic dysfunction in type 2 diabetes mellitus. J Am Coll Cardiol.

[55] Bakermans AJ, Geraedts TR, van Weeghel M, Denis S, Ferraz MJ, Aerts JMFG, Aten J, Nicolay K, Houten SM, Prompers JJ (2011). Fasting-induced myocardial lipid accumulation in long-chain acyl-CoA dehydrogenase knock-out mice is accompanied by impaired left ventricular function. Circ Cardiovasc Imaging.

[56] Bilheimer DW, Buja LM, Parkey RW, Bonte FJ, Willerson JT (1978). Fatty acid accumulation and abnormal lipid deposition in peripheral and border zones of experimental myocardial infarcts. J Nucl Med.

[57] Jodalen H, Stangeland L, Grong K, Vik-Mo H, Lekven J (1985). Lipid accumulation in the myocardium during acute regional ischaemia in cats. J Mol Cell Cardiol.

[58] Evanochko WT, Reeves RC, Sakai TT, Canby RC, Pohost GM (1987). Proton NMR spectroscopy in myocardial ischemic insult. Magn Reson Med.

[59] Bouchard A, Doyle M, Wolkowicz PE, Wilson R, Evanochko WT, Pohost GM (1991). Visualization of altered myocardial lipids by 1H NMR chemical-shift imaging following ischemic insult. Magn Reson Med.

[60] Straeter-Knowlen IM, Evanochko WT, den Hollander JA, Wolkowicz PE, Balschi JA, Caulfield JB, Ku DD, Pohost GM (1996). 1H NMR spectroscopic imaging of myocardial triglycerides in excised dog hearts subjected to 24 hours of coronary occlusion. Circulation.

[61] Reeves RC, Evanochko WT, Canby RC, McMillin JB, Pohost GM (1989). Demonstration of increased myocardial lipid with postischemic dysfunction (“myocardial stunning”) by proton nuclear magnetic resonance spectroscopy. J Am Coll Cardiol.

[62] Ingwall JS, Weiss RG (2004). Is the failing heart energy starved? On using chemical energy to support cardiac function. Circ Res.

[63] Beer M, Seyfarth T, Sandstede J, Landschütz W, Lipke C, Köstler H, Von Kienlin M, Harre K, Hahn D, Neubauer S (2002). Absolute concentrations of high-energy phosphate metabolites in normal, hypertrophied, and failing human myocardium measured noninvasively with (31)P-SLOOP magnetic resonance spectroscopy. J Am Coll Cardiol.

[64] Bottomley PA, Herfkens RJ, Smith LS, Brazzamano S, Blinder R, Hedlund LW, Swain JL, Redington RW (1985). Noninvasive detection and monitoring of regional myocardial ischemia in situ using depth-resolved 31P NMR spectroscopy. Proc Natl Acad Sci USA.

[65] Caus T, Izquierdo M, Lan C, Le Fur Y, Confort-Gouny S, Cozzone PJ, Bernard M (2001). Simultaneous study of metabolism and function following cardioplegic arrest: a novel method of evaluation of the transplanted heart in the rat. J Heart Lung Transpl.

[66] Neubauer S, Horn M, Cramer M, Harre K, Newell JB, Peters W, Pabst T, Ertl G, Hahn D, Ingwall JS, Kochsiek K (1997). Myocardial phosphocreatine-to-ATP ratio is a predictor of mortality in patients with dilated cardiomyopathy. Circulation.

[67] Xiong Q, Ye L, Zhang P, Lepley M, Swingen C, Zhang L, Kaufman DS, Zhang J (2012) Bioenergetic and functional consequences of cellular therapy: activation of endogenous cardiovascular progenitor cells. Circ Res 11:455–468 10.1161/CIRCRESAHA.112.269894 PMC3635674 22723295

[68] Bottomley PA, Weiss RG (2001). Noninvasive localized MR quantification of creatine kinase metabolites in normal and infarcted canine myocardium. Radiology.

[69] Ingwall JS, Shen W (1999). The chemistry of ATP in the failing heart the fundamentals. Heart Fail Rev.

[70] Nakae I, Mitsunami K, Yabe T, Inubushi T, Morikawa S, Matsuo S, Koh T, Horie M (2004). Detection of metabolic abnormality in asynergic regions of ischemic human myocardium using 31P and 1H magnetic resonance spectroscopy. J Cardiovasc Magn Reson.

[71] Nakae I, Mitsunami K, Omura T, Yabe T, Tsutamoto T, Matsuo S, Takahashi M, Morikawa S, Inubushi T, Nakamura Y, Kinoshita M, Horie M (2003). Proton magnetic resonance spectroscopy can detect creatine depletion associated with the progression of heart failure in cardiomyopathy. J Am Coll Cardiol.

[72] Nakae I, Mitsunami K, Matsuo S, Matsumoto T, Morikawa S, Inubushi T, Koh T, Horie M (2004). Assessment of myocardial creatine concentration in dysfunctional human heart by proton magnetic resonance spectroscopy. Magn Reson Med Sci.

[73] Nakae I, Mitsunami K, Matsuo S, Inubushi T, Morikawa S, Tsutamoto T, Koh T, Horie M (2005). Myocardial creatine concentration in various nonischemic heart diseases assessed by 1H magnetic resonance spectroscopy. Circ J.

[74] Shen W, Asai K, Uechi M, Rathier MA, Shannon RP, Vatner SF, Ingwall JS (1999). Progressive loss of myocardial ATP due to a loss of total purines during the development of heart failure in dogs: a compensatory role for the parallel loss of creatine. Circulation.

[75] Shen W, Vatner DF, Vatner SF, Ingwall JS (2010). Progressive loss of creatine maintains a near normal Δ*G* ~ ATP in transgenic mouse hearts with cardiomyopathy caused by overexpressing Gsα. J Mol Cell Cardiol.

[76] Horn M, Frantz S, Remkes H, Laser A, Urban B, Mettenleiter A, Schnackerz K, Neubauer S (1998). Effects of chronic dietary creatine feeding on cardiac energy metabolism and on creatine content in heart, skeletal muscle, brain, liver and kidney. J Mol Cell Cardiol.

[77] Wallis J, Lygate CA, Fischer A, ten Hove M, Schneider JE, Sebag-Montefiore LM, Dawson D, Hulbert K, Zhang W, Zhang MH, Watkins H, Clarke K, Neubauer S (2005). Supranormal myocardial creatine and phosphocreatine concentrations lead to cardiac hypertrophy and heart failure: insights from creatine transporter-overexpressing transgenic mice. Circulation.

[78] ten Hove M, Chan S, Lygate CA, Monfared M, Boehm EA, Hulbert K, Watkins H, Clarke K, Neubauer S (2005). Mechanisms of creatine depletion in chronically failing rat heart. J Mol Cell Cardiol.

[79] Phillips D, ten Hove M, Schneider JE, Wu CO, Sebag-Montefiore LM, Aponte AM, Lygate CA, Wallis J, Clarke K, Watkins H, Balaban RS, Neubauer S (2010). Mice over-expressing the myocardial creatine transporter develop progressive heart failure and show decreased glycolytic capacity. J Mol Cell Cardiol.

[80] ten Hove M, Lygate CA, Fischer A, Schneider JE, Sang AE, Hulbert K, Sebag-Montefiore LM, Watkins H, Clarke K, Isbrandt D, Wallis J, Neubauer S (2005). Reduced inotropic reserve and increased susceptibility to cardiac ischemia/reperfusion injury in phosphocreatine-deficient guanidinoacetate-N-methyltransferase-knockout mice. Circulation.

[81] Weiss K, Martini N, Boesiger P, Kozerke S (2012) Metabolic MR imaging of regional triglyceride and creatine content in the human heart. Magn Reson Med. doi:10.1002/mrm.24178 10.1002/mrm.24178 22294511

